# Morning Anaerobic Performance Is Not Altered by Vigilance Impairment

**DOI:** 10.1371/journal.pone.0058638

**Published:** 2013-03-13

**Authors:** Romain Lericollais, Antoine Gauthier, Nicolas Bessot, Amira Zouabi, Damien Davenne

**Affiliations:** 1 Normandie Université, Caen, France; 2 UniCaen, unité de recherche COMETE, Caen, France; 3 Institut national de la santé et de la recherche médicale, unité de recherche U1075 COMETE, Caen, France; 4 Centre Hospitalier Universitaire de Caen, Department of Clinical Physiology, Caen, France; St. Joseph's Hospital and Medical Center, United States of America

## Abstract

The aim of this study was to determine the role played by vigilance on the anaerobic performance recorded during a Wingate test performed at the bathyphase (nadir) of the circadian rhythmicity. Twenty active male participants performed a 60-s Wingate test at 6 a.m. during 3 test sessions in counter-balanced order the day after either (i) a normal reference night, (ii) a total sleep deprivation night, or (iii) a total sleep deprivation night associated with an extended simulated driving task from 9 p.m. to 5 a.m. During this task, the number of inappropriate line crossings (ILCs) was used to control and quantify the effective decrease in the level of vigilance. The main findings show that (i) vigilance of each participant was significantly altered (i.e., a drastic and progressive increase in ILCs is shown during the 7.5 hours of driving) by the sleep deprivation night associated with an extended driving task; (ii) the subjective evaluation of vigilance performed by self-rated scale revealed an increased impairment of the vigilance level between the normal reference night, the total sleep deprivation night and the total sleep deprivation night associated with an extended driving task; and (iii) the morning following this last condition, during the Wingate test, the recorded cycling biomechanical parameters (peak power, mean power and fatigue index values, power decrease, and cycling kinetic and kinematic patterns) were not significantly different from the two other conditions. Consequently, these results show that anaerobic performances recorded during a Wingate test performed at the bathyphase of the circadian rhythmicity are not altered by a drastic impairment in vigilance. These findings seem to indicate that vigilance is probably not a factor that contributes to circadian variations in anaerobic performance.

## Introduction

Anaerobic performances obtained during a Wingate test fluctuate as a function of the time of day [1]–[6]. Both peak power (PP) and mean power (MP) recorded throughout the circadian cycle show acrophase (peak time) and bathyphase (trough time or nadir), which are observed at about 6 p.m. and 6 a.m., respectively, with an amplitude (half of the peak-to-trough variation) amounting to 7.6% for PP and 11.3% for MP [6].

The Wingate test consists of a cycling exercise that involves a specific movement showed to be pluri-axial, pluri-articular, and requiring several muscular chains at a free pedal rate. This movement also involves motor coordination based on a regulation of the different biomechanical patterns and dependent on the optimal functional state of nervous system, and could be analyzed thanks to recordings of the cycling kinetic and kinematic patterns [7]–[10]. For these reasons, the Wingate test is considered as a complex exercise which involves several physiological and biomechanical factors determining the performance [11], [12]. Most of these factors are clearly affected by the time of day [2], [5], [6], [13]–[15]. They fluctuate in phase with the power output values (PP and MP) recorded during the Wingate test throughout the day and this could explain the diurnal variations in anaerobic performances [1]–[6].

Cognitive factors such as motivation, arousal, and/or vigilance are also involved in the performance obtained during the Wingate test [11], [12] and have been shown to explain the diurnal fluctuations in anaerobic performances [1], [4], [6], [16]. Among these, it has been found that vigilance is impaired in the morning in comparison with the evening [16], [17]. Vigilance is associated with a particular state of cortical activation, while a decline in vigilance is characterized by changes in cortical activation levels [18]. These changes result in the incapacity of the central nervous system to sustain a performance or mental effort. The variations described in anaerobic power output values obtained during the Wingate test throughout the day could be related to the concomitant diurnal fluctuations in vigilance level [1], [4], [6]. However, to the best of our knowledge, a causal link between the diurnal variations in vigilance and anaerobic performance has never been demonstrated.

To check the effects of the diurnal fluctuations in vigilance on the anaerobic performance during the day, it is required to induce a variation of the vigilance level (impairment or improvement) applied at one of the two main points of its circadian rhythm, i.e., at the bathyphase or the acrophase. Methodologically, it is easier to impair the vigilance than to improve it. Thereby, most of the chronobiological studies which aim to study the effects of vigilance applied such an impairment. Then, sleep deprivation is the appropriate experimental condition to induce this impairment. Furthermore, an evaluation performed immediately after this impairment is needed because it is more controllable and less restrictive for the participants than an evaluation performed several hours later. According to these criteria, in the literature, only the study by Souissi *et al.* [19] was selected. It showed that there was no effect of one night of total sleep deprivation on power output values recorded during a 30-s Wingate test the following day at 6 a.m. However, in this study, the decrease in vigilance was not controlled and it is unknown to what extent this parameter has been affected.

To efficiently test the influence of vigilance on the anaerobic performance measured during a Wingate test, an experimental condition inducing an effective vigilance impairment, which is controlled and quantified, is required. To that purpose, a monotonous and prolonged nocturnal driving task has been recently recognized to drastically impair vigilance [20], [21]. The use of a special fixed-based driving simulator, as that used in the Davenne *et al.* [20] study, appears to be suitable to objectively quantify and control the vigilance level during the night of total sleep deprivation. In this context, if a causal link exists between the diurnal variations in vigilance and those of anaerobic performance, it can be hypothesized that impairment in vigilance would decrease the performance observed during sustained anaerobic cycling exercise at 6 a.m.

The aim of this study was to determine the role played by vigilance on the anaerobic performance recorded during a Wingate test performed at the bathyphase (nadir) of the circadian rhythmicity.

## Materials and Methods

### Ethics Statement

The experimental protocol and the procedure for obtaining informed consent were approved by the local ethics committee (Comité Consultatif de Protection des Personnes qui se prêtent à des Recherches Biomédicales du CHU de Caen, France) and the study complied with the requirements for the conduct of biological rhythm research on human beings [22] and the tenets of the Declaration of Helsinki. Prior to participating, every participant provided written informed consent once the procedures were explained in detail.

### Participants

Twenty active male participants (mean ± SEM; age: 21.2±0.3 years; height: 178.7±1.3 cm; weight: 70.3±2.3 kg) not specially trained in cycling and adhering to a routine of diurnal activity between 6∶30 a.m. to 7∶30 a.m and 10∶30 p.m. to 11∶30 p.m. alternating with night-time sleep, volunteered to participate in this investigation. All participants were selected as “neither type”, on the basis of their answers to the Horne & Östberg [23] self-assessment questionnaire, which determines morningness-eveningness. Following this questionnaire, core body temperature, considered as a chronobiological marker, i.e., representative of the functioning in the circadian system [24], was recorded for each participant continuously every 60 s during a 24-h period. It was recorded by means of a telemetric monitoring system (VitalSense®, Mini Mitter Co. Inc., Bend, OR, USA), consisting of a thermistor-based ingestible and biocompatible capsule (gastrointestinal (GI) temperature sensor) and receiver/monitor [25]. To ensure that the temperature sensor is further down the GI tract, it was swallowed at least 4 h before the beginning of the recordings [26]. As GI temperature can be influenced by water and food intake or by physical exercise (for a review, see Lim et al. [26]), participants were asked to note the clock time of these events in a notebook to suppress spurious data in the recordings.

### Experimental Procedure

**Figure 1:**
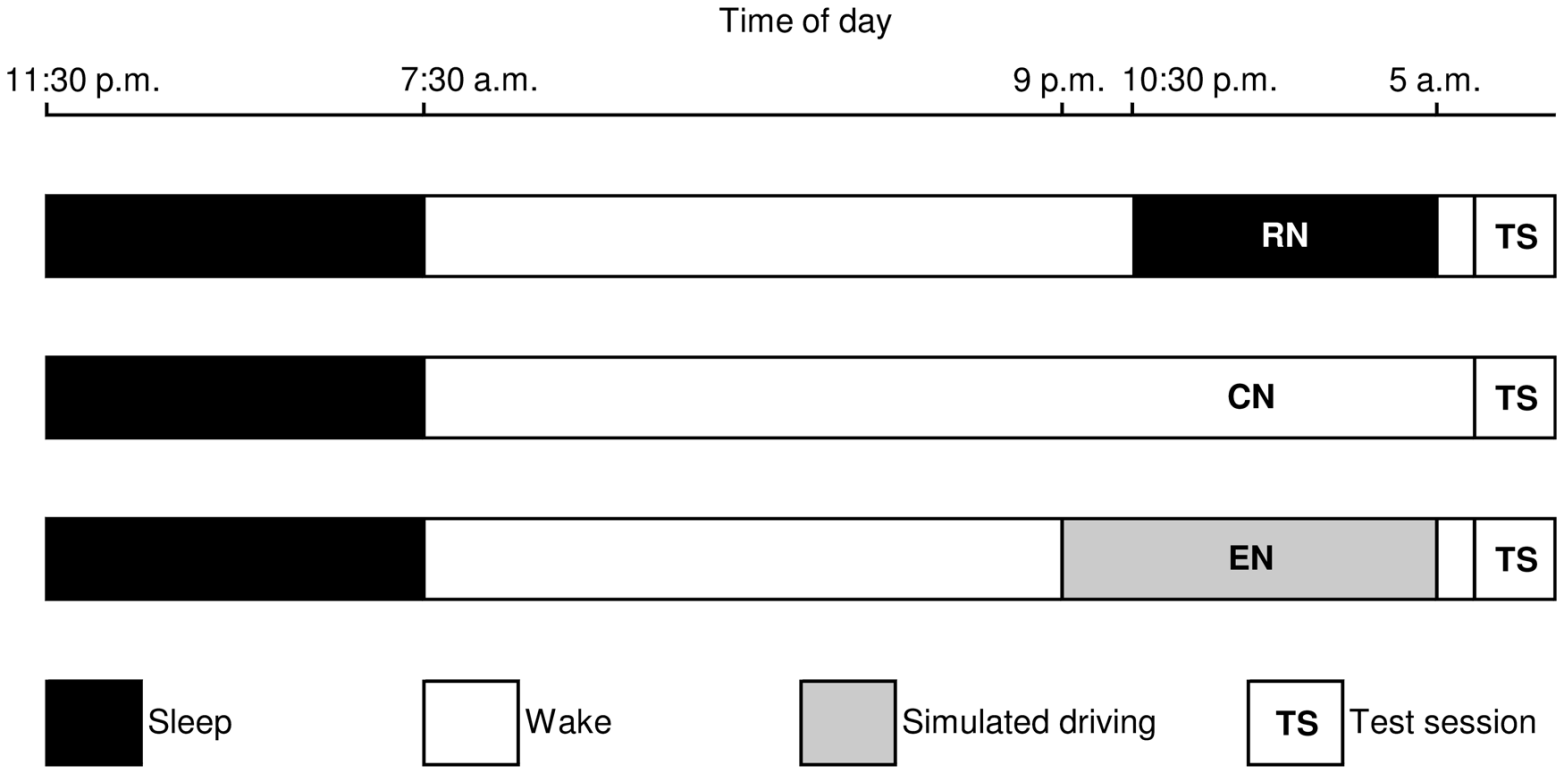
Design of the experimental protocol. The sleep-wake periods and the three test sessions performed after a reference night (RN), a control night (CN), and an experimental night (EN) are shown.

During an initial session of habituation carried out one week before the start of the testing period, all participants were familiarized with the test procedure. The testing period began 24 h prior to the first test session with the implementation of an actimeter, and ended after the third test session. Participants underwent three test sessions at 6 a.m. (Greenwich Mean Time +1 h) the day following: (i) a normal reference night (RN) during which the participants had to sleep between 10∶30 p.m. and 5 a.m. (mean sleep duration was 6 h 06 min ±3 min); (ii) a total sleep deprivation night without activities (control night or CN); and (iii) a total sleep deprivation night associated with a sustained attention psychomotor task (experimental night or EN) (Figure 1). The timing of the three test sessions, 6 a.m., adheres to the expected minima of the circadian rhythms in anaerobic performance and core temperature for the selected population [6], [14]. The three test sessions were performed in a counter-balanced order with at least one week between each test session. During the 24 h before each test session, participants were prohibited from eating or drinking any stimulant, e.g., caffeine or any other ergogenic drug that could enhance wakefulness. Participants did not engage in any tiring exercise during the testing period, and they were instructed to adhere as closely as possible to their usual sleeping habits for at least one week before the start of the testing period. For each participant, the sleep duration and daily activity throughout the experiment were continuously recorded and quantified by actimetry (Actiwatch®, Neurotechnology, Cambridge, UK). This device monitors body movement of the participant by means of an actigraph placed on the wrist of the non-dominant arm. The collected data were checked in order to be sure that the participants adhered to the instructions (e.g., bed and waking times and activity).

Before the RN test session, participants were instructed to go to bed at about 10∶30 p.m. and to wake up at 5 a.m. (Figure 1); at this time, they were allowed to drink only one glass of water as recommended by Bougard *et al.* [27], [28].

For both CN and EN test sessions, participants came to the laboratory at 8∶30 p.m., and were not allowed to sleep thereafter until 6 a.m. (Figure 1). Participants were supervised by two experimenters. They could drink water at any time, and at 1 a.m. they were allowed to eat a cereal bar that contained approximately 830 kJ. To stay awake during a CN, participants could read books, do their homework or listen to music. Also, they were instructed to change activities regularly during the night.

During an EN, participants performed a psychomotor task requiring sustained attention [20], [21], which consisted of simulated driving for 7.5 hours from 9 p.m. to 5 a.m. [20] (Figure 1). During this driving simulation, participants were instructed to use the steering wheel to maintain the vehicle in the middle of the road, and not to cross the painted lines separating the lanes during eight identical trips interrupted by a break of few minutes in the course of which the participants were allowed to drink one glass of water and/or go to the toilet. During the driving test, participants were supervised by one experimenter. The simulated driving task took place in a part of the laboratory in which the temperature (20.8±0.2°C), relative humidity (36.3±1.5%), and luminosity (100 lux) remained constant.

### Bicycle Ergometer Test

For each test session at 6 a.m., participants were asked to perform a sustained Wingate test on a friction-loaded cycle ergometer (Monark 874E, Varberg, Sweden) equipped with pedals with foot-straps. This test appears to be particularly appropriate to evaluate the effects of vigilance on anaerobic performance and fatigue phenomenon. Furthermore, it is recognized to be a reproducible, reliable and valid test [11], [12]. This anaerobic cycling test consisted of cycling as fast as possible for 60 s against a constant braking load dependent on the body mass of the participant (0.075 kg/kg body mass) according to the optimization tables of Bar-Or [11]. For each participant, the braking load was determined during the first test session and remained constant throughout the experiment. The cycle ergometer was designed to apply a load in increments of 0.1 kg. Therefore, each applied individual load was rounded off to the closest value. The duration of the test was extended to its maximum for an optimal assessment of the anaerobic processes, according to Vandewalle *et al.* [12] and Withers *et al.* [29], [30]. At the beginning of the Wingate test, participants had to remain in a stationary position, and at the start signal, participants began pedaling seated on the saddle. To avoid the intra-individual effect of postural changes on cycling performance and biomechanical variables [31]–[33], the foot position on the pedals, saddle height, and upper body position of each participant were recorded and maintained identical during the three test sessions. Throughout each trial, participants were instructed to keep their hands in pronation on the lower part of the handlebar and to remain in a sitting position. Strong verbal encouragements were given to each participant by the same person throughout the entire test to help motivate the participant to develop his maximal power output until the very end of the 60-s test. Verbal indications were also given on the time count, every 10 s, until the end of the exercise.

### Data Collection and Analysis

#### Objective vigilance and subjective sleepiness and fatigue

During the simulated driving task, vigilance was objectively evaluated by analyzing the number of inappropriate line crossings (ILCs) recorded during each simulated highway trip [20], [21]. In addition, at the end of each simulated highway trip, participants were asked to complete the Karolinska Sleepiness Scale (KSS) [34] to estimate their subjective sleepiness. KSS scale ranges from 1 to 9 (“very alert” to “very sleepy, fighting sleep, an effort to keep awake”).

Before each physical test session at 6 a.m., participants were asked to complete the KSS and evaluate their subjective fatigue on a 100 mm visual analogical scale (VAS) (“Describe how fatigued you are now”) ranging between “not at all tired” and “very tired”.

#### Wingate-test-related data

At the beginning of each test session, the body mass of each participant was measured using a digital scale (Tefal® Sensio, Ecully, France).

The cycle ergometer was equipped with the SRM PowerMeter Science system (Schoberer Rad Messtechnik, Jülich, Germany) which consists of the crank, chainrings, and measuring unit. During each test session, power output was continuously monitored at each pedaling cycle. The power output values (W) were recorded and averaged for samples of 2 s. In this study, three classic parameters of the Wingate test were analyzed: (i) the Peak Power (PP), which is the highest mechanical power attained during the course of the exercise; (ii) the Mean Power over 30 s (MP_30s_) and over 60 s (MP_60s_), corresponding, respectively, to the average of the power output values obtained during the first 30 s and during the full 60 s of the test; and (iii) Fatigue Index (FI), expressed in percentage (%), which is calculated from the difference between PP and the minimal power output divided by PP [5] and it was used to investigate the global power decrease during the test. Furthermore, the decrease in 30 averaged power output values throughout the 60-s Wingate test was used to analyze the power decrease.

During the anaerobic fatigue cycling test, kinetic and kinematic variables were continuously measured during two 15-s intervals of the sustained Wingate test [2]: the first interval (I_1_) from the 5^th^ to the 20^th^ second and the second interval (I_2_) from the 45^th^ to the 60^th^ second.

Kinetic data, corresponding to the evolution of instantaneous torque developed to the crank axis throughout a complete pedal cycle, were recorded by eight strain gauges located between the crank arm and the chainrings of the SRM system at a sampling frequency of 200 Hz. Kinetics was analyzed using the method described by Bessot *et al.* [35] for a cycling movement. Torque (Nm) reflected the ratio between the effective force (i.e., the propulsive force applied perpendicularly to the crank arm) and the constant length of the crank arm. The average curve illustrating the evolution of instantaneous torque throughout a complete revolution of the crank was automatically computed by the mean of the values collected during each 15-s recording. The evolution of instantaneous torque throughout a complete revolution of the crank reached two peaks and two low points that, respectively, fit with: (i) the maximum propulsive work reached during each leg extension phase; and (ii) the top- and bottom-dead-center. The data reflecting the left leg (from 0° to 180°; 0° corresponding to the crank when the left pedal is at the top) and the right leg (from 180° to 360°) contributions were averaged. From the average curve, two parameters were analyzed: (i) the angle of peak torque; and (ii) the range in torque variation were determined for each 15-s interval. The angle of peak torque corresponds to the crank angle when the peak torque is observed and allows us to explore the torque phase shift. We followed the method of Bessot *et al.* [35], in which a sinusoidal function using regression analysis was fitted to the mean absolute torque values because, in some participants, the absolute data reached a plateau around the peak torque making the detection of the peak torque angle impossible. The sinusoidal function was expressed as follows:
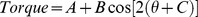
where *θ* is the crank angle, and *A*, *B* and *C* are constants to be determined. The *C* value was determined from the sine function fit to the crank angle of peak torque, and was used to analyze the angle of peak torque shift. The crank angle is the angle between the vertical axis (0°) passing through the crank axis, and the crank arm. The range in torque variation is determined by calculating the difference between minimum and maximum torque values and is expressed in Nm.

Kinematic variables of pedaling movement were measured using an optoelectronic system (Kinemetrics®, MIE medical research, Ltd., Leeds, UK) consisting of three infrared cameras (sampling frequency 50 Hz), which kept track of five reflective markers during cycling. In order to model the pedaling cycle, four markers were placed on the right lower limb of the participants (trochanter major, *epicondylus lateralis femoris, malleolus lateralis*, and the big toe) and one marker was fixed on the pedal axis.

For kinematic variables, three lower limb joint angles were studied, i.e., the angles of the hip, knee, and ankle. Hip angle is formed by two anatomical axes: *trochanter major*/*epicondylus lateralis femoris* and the horizontal axis. Knee angle consists of two axes: *trochanter major*/*epicondylus lateralis femoris* and *epicondylus lateralis femoris*/*malleolus lateralis*. Ankle angle corresponds to the angle between two axes: *epicondylus lateralis femoris*/*malleolus lateralis* and *malleolus lateralis*/big toe. To analyze kinematic data, the angles of each joint were measured and averaged every 22.5° across the crank cycle. Only the complete crank cycles recorded during each 15-s interval were taken into account. The mobilization of these three joints was characterized by studying two parameters: (i) the mean angle, which is the average of angles recorded during the complete pedaling cycle; and (ii) the mean range of motion (ROM), which corresponds to the difference between the minimum and maximum joint angles collected throughout the pedal cycle. The mean angle and ROM refer to the average of cycles recorded during 15 s. This procedure was conducted during each 15-s interval for hip, knee, and ankle angles [2].

### Statistical Analysis

Statistical tests were processed using SigmaStat 3.5 Software (SYSTAT, Point Richmond, CA, USA). The data distribution normality was automatically tested (Kolmogorov-Smirnov test). All values are expressed as mean ± SEM.

To identify the circadian rhythm and to demonstrate that the rhythm amplitude differed from 0, core temperature data were subjected to a one-way analysis of variance (ANOVA) for repeated measures with the factor ‘‘time of day’’. A least-squares linear regression analysis using cosinor analysis was used to determine the best fit of a 24-h period cosine function [36]:


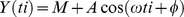
, where *M* is the MESOR (Medline Estimating Statistic Of Rhythm which corresponds to the estimated mean level of the rhythm), *A* is the amplitude (equal to 0.5 of the peak-to-trough variation due to rhythmicity), and *φ* is the acrophase (clock time at the maximal level in circadian rhythm). The bathyphase (clock time at the minimal level in circadian rhythm) occurs 12 hours before the acrophase. The existence of a sinusoidal circadian rhythm was confirmed or rejected on the basis of the 95% confidence interval.

During EN, the impaired driving performance and the subjective sleepiness were analyzed using a one-way analysis of variance (ANOVA) with repeated measures (“driving duration”) applied to the mean values of ILCs recorded during each of eight simulated highway trips and the mean values of self-rated sleepiness scores recorded after each trip, respectively. When significant effects were detected by ANOVA, a post hoc analysis (Tukey’s test) was performed to compare the mean values recorded during each trip for the driving data and after each trip for the subjective sleepiness data, respectively. Pearson’s correlation coefficient was calculated to determine relationships between the subjective sleepiness scores and the ILCs during the nocturnal simulated driving task.

A one-way analysis of variance (ANOVA) with repeated measures using the factor “test session” was used to identify the effect of RN, CN, and EN on the mean values of self-rated fatigue and sleepiness scores, PP, MP_30s_, MP_60s_, and FI. To evaluate the effect of RN, CN, and EN on power decrease, a two-way analysis of variance (ANOVA) with repeated measures (“test session”×“time points across the Wingate test”) was applied to the absolute power output values obtained every 2 s throughout the entire test. For each 15-s interval (I_1_ and I_2_), the mean values of angle of peak torque, absolute range in torque variation, mean angle, and ROM of the three studied joints were submitted to a one-way analysis of variance (ANOVA) for repeated measures with the factor “test session”. When significant effects were detected by ANOVA, a post hoc analysis (Tukey’s test) was performed to compare the mean values recorded at 6 a.m. RN, CN and EN. Statistical significance was accepted at *p*<0.05.

## Results

### Core Body Temperature

**Figure 2:**
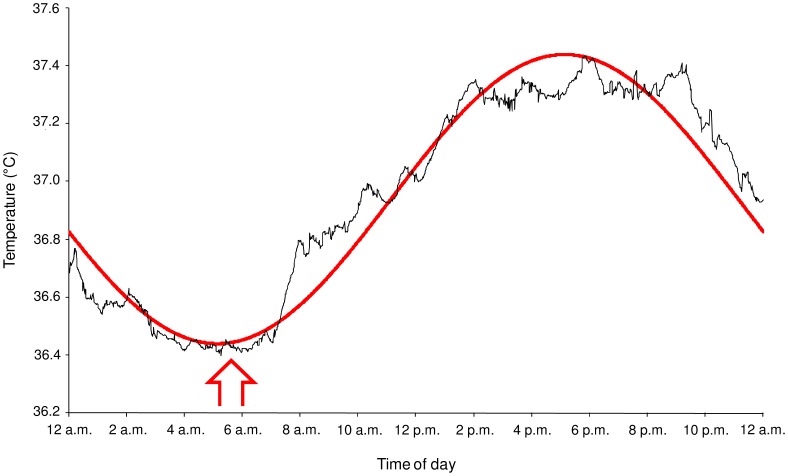
Circadian rhythm of gastrointestinal temperature. Mean values (black line) recorded every 60 s during 24-h period are shown. The mean best-fit curve (red line) between the experimental data and the cosine curve of the 20 participants is shown (*r* = 0.97). Red vertical arrow illustrates the beginning of the three morning test sessions.

A circadian rhythm was found in GI temperature (*p*<0.001). The mean fitted cosine curve for a fixed period of 24 h (*r* = 0.97; *p*<0.001) obtained from that of the 20 participants indicated a temperature acrophase at 5∶08±0∶19 p.m., a bathyphase at 5∶08±0∶19 a.m., and a MESOR of 36.94±0.08°C. The peak-to-trough amplitude of the rhythm was 1.0±0.04°C (Figure 2).

### Objective Vigilance and Subjective Sleepiness and Fatigue

**Figure 3:**
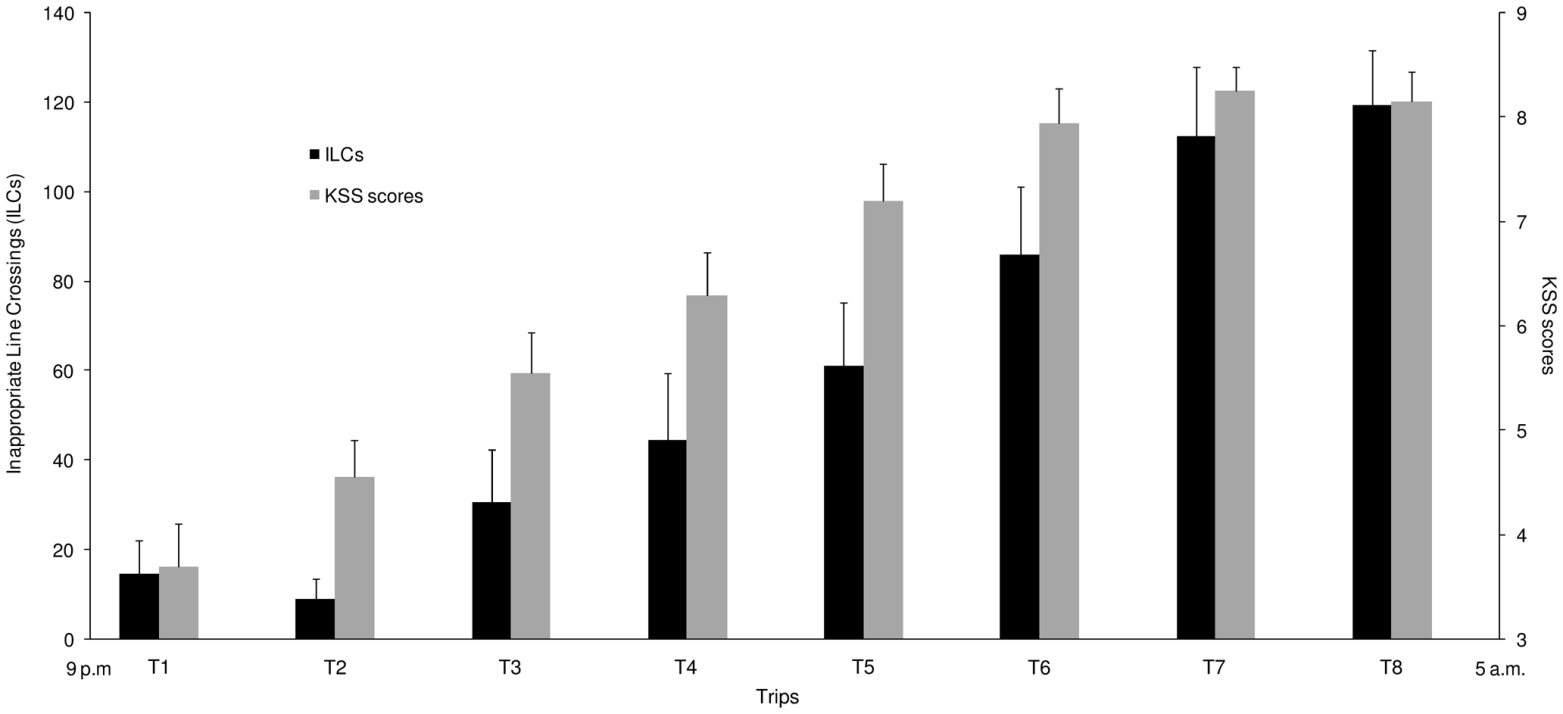
Follow up of ILCs and KSS scores recorded during the nocturnal simulated driving task. Data are mean ± SEM (*n* = 20).

The one-way ANOVA showed a “driving duration” effect on ILCs (F_(7,133)_ = 27.11; *p*<0.001) characterized by a significant increase in ILCs as a function of simulated driving duration (Figure 3). The post hoc analysis revealed that mean values of ILCs observed during the first 3 trips are not significantly different and a progressive increase in ILCs was detected until the trips 7 and 8 where the ILCs were the highest. When comparing the first and last trips, the ILCs reported during the trip 8 (119.5±12.3) were drastically increased (*p*<0.001) compared to the mean values of ILCs recorded during trip 1 (14.5±7.5) (Figure 3).

The one-way ANOVA showed a “driving duration” effect on subjective sleepiness scores (F_(7,133)_ = 57.92; *p*<0.001) characterized by a significant increase in scores throughout the simulated driving task (Figure 3). When comparing the first and last trips, the sleepiness scores reported after the trip 8 (8.1±0.3) were drastically increased (*p*<0.001) compared to the mean values of scores recorded after trip 1 (3.7±0.4) (Figure 3). Subjective sleepiness scores were positively correlated with ILCs (*r* = 0.61; *p*<0.001).

The self-rated sleepiness scores recorded before the sustained Wingate test session at 6 a.m. were significantly different between the three test sessions (F_(2,38)_ = 55.61; *p*<0.001). The sleepiness score of the EN (8.1±0.3) was higher than the CN (7.0±0.2) and the RN (3.6±0.4) scores (*p*<0.05 and *p*<0.001, respectively). The sleepiness score of the CN was higher than the RN score (*p*<0.001).

A significant difference on the self-rated fatigue scores was also detected between the three test sessions (F_(2,38)_ = 29.45; *p*<0.001). The fatigue score of the EN (69.8±3.4 mm) was higher than the CN (58.8±3.5 mm) and the RN (40.9±2.4 mm) scores (*p*<0.05 and *p*<0.001, respectively). The fatigue score of the CN was higher than the RN score (*p*<0.001).

### Cycling Biomechanical Parameters During the Wingate Test

#### Peak power, mean power and fatigue index

No significant difference was found in PP (F_(2,38)_ = 2.23; p = 0.12), MP_30s_ (F_(2,38)_ = 0.75; p = 0.48), MP_60s_ (F_(2,38)_ = 0.17; p = 0.84) and FI (F_(2,38)_ = 2.75; p = 0.08) between RN, CN, and EN (Table 1).

**Table 1:** Anaerobic power output values and Fatigue Index (mean ± SEM) obtained during the 60-s Wingate test performed the following morning after 3 different nights.

	RN	CN	EN
PP (W)	799.7±26.1	824.2±24.8	809.0±25.2
MP_30s_ (W)	593.9±15.9	596.1±15.3	591.5±15.0
MP_60s_ (W)	454.2±10.7	452.8±10.7	452.8±10.1
FI (%)	69.2±0.9	71.2±1.2	70.7±1.2

PP, Peak Power; MP_30s_ and MP_60s_, Mean Power recorded during the first 30 s and the full 60 s of the test, respectively; FI: Fatigue Index; RN, test session after a normal reference night; CN, test session after a total sleep deprivation night; EN, test session after a total sleep deprivation night associated with 7.5 h of simulated driving (*n* = 20).

#### Power decrease

**Figure 4:**
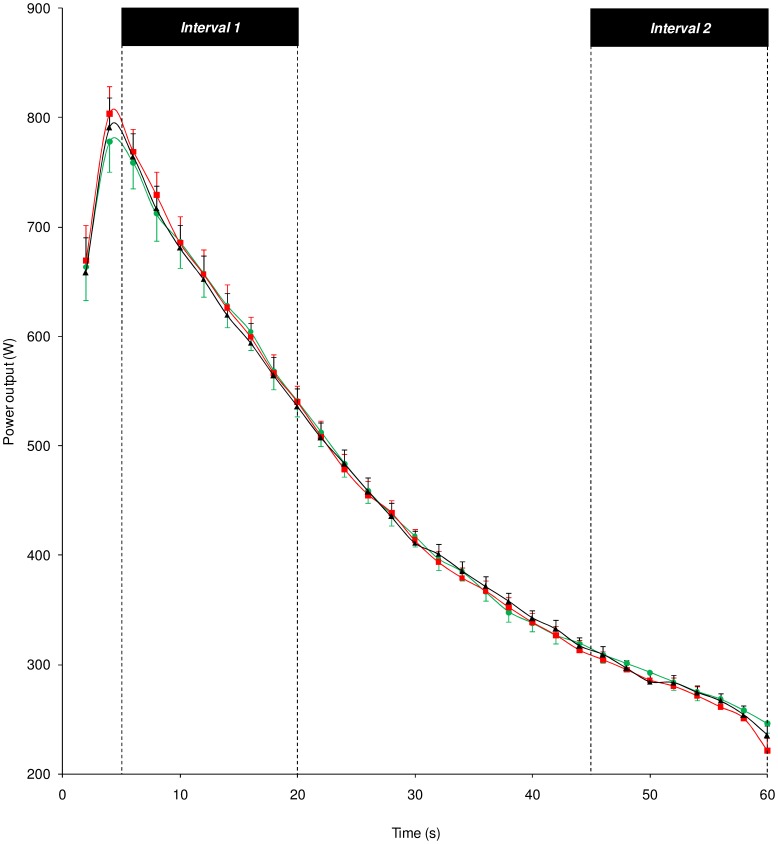
Evolution of power output values recorded throughout the 60-s Wingate test. Evolution of power output values as a function of time points throughout the 60-s Wingate test after a reference night or RN (green symbols), a control night or CN (red symbols) and an experimental night or EN (black symbols). Intervals 1 and 2 during which kinetic and kinematic variables were recorded and analyzed are shown. Data are mean ± SEM (*n* = 20).

The two-way ANOVA revealed no “test session” effect on power outputs recorded the day following the three types of night (F_(2,38)_ = 0.17; p = 0.84). There was a “time points across the Wingate test” effect (F_(29,551)_ = 294.43; p<0.001) characterized by a significant decrease in power output values throughout the 60-s trial during the three test sessions. No “test session”×“time points across the Wingate test” interaction (F_(58,1102)_ = 0.86; p = 0.77) was detected in power output value decreases (Figure 4).

#### Kinetic data

The angle of peak torque did not vary significantly between the three test sessions, neither during I_1_ (F_(2,38)_ = 3.09; p = 0.06), nor during I_2_ (F_(2,38)_ = 1.68; p = 0.20) (Table 2).

**Table 2:** Kinetic variables: Angle of peak torque and range in torque variation (mean ± SEM) recorded over a complete crank cycle during interval 1 (I_1_) and interval 2 (I_2_) of the Wingate test performed the following morning after 3 different nights.

	I_1_			I_2_		
	RN	CN	EN	RN	CN	EN
Angle of peak torque (°)	90.7±4.8	98.3±4.8	90.1±3.9	96.3±1.0	97.0±1.0	97.7±1.1
Range in torque variation (Nm)	39.6±1.9	37.3±2.5	39.2±2.4	68.4±3.6	67.4±3.7	68.5±3.6

RN, test session after a normal reference night; CN, test session after a total sleep deprivation night; EN, test session after a total sleep deprivation night associated with 7.5 h of simulated driving (*n* = 20).

During I_1_ (F_(2,38)_ = 2.11; *p = *0.14) and I_2_ (F_(2,38)_ = 0.84; *p = *0.44), the absolute mean values of the range in torque variation measured during a complete crank cycle were not significantly different between the three test sessions (Table 2).

#### Hip, knee, or ankle mobility

During I_1_ and I_2_, the comparison between RN, CN, and EN showed no significant difference for the mean angles of the hip (F_(2,38)_ = 1.39; p = 0.26 and F_(2,38)_ = 0.98; p = 0.39, respectively), the knee (F_(2,38)_ = 0.19; p = 0.83 and F_(2,38)_ = 0.09; p = 0.91, respectively) or the ankle (F_(2,38)_ = 1.38; p = 0.26 and F_(2,38)_ = 0.54; p = 0.59, respectively) recorded throughout the complete pedaling cycle (Table 3).

**Table 3:** Kinematic variables: Mean angle and ROM (mean ± SEM) of the hip, knee and ankle angles measured throughout the complete crank cycle during interval 1 (I_1_) and interval 2 (I_2_) of the Wingate test performed the following morning after 3 different nights.

		Mean angle (°)			ROM (°)		
		RN	CN	EN	RN	CN	EN
Hip	I_1_	38.2±0.8	38.0±0.8	37.5±0.8	43.2±0.6	43.4±0.6	43.5±0.6
	I_2_	40.4±0.8	40.0±0.8	39.5±1.0	40.7±0.9	40.5±0.8	41.2±1.0
Knee	I_1_	103.9±1.1	103.7±1.0	103.4±1.2	71.4±1.0	71.2±0.9	70.9±1.1
	I_2_	104.7±1.0	104.3±0.8	104.4±1.1	74.4±1.7	73.2±1.4	72.8±1.8
Ankle	I_1_	110.1±2.3	107.5±1.5	106.8±1.5	21.2±1.1	20.2±1.1	20.5±1.1
	I_2_	100.2±1.9	101.1±2.0	101.0±2.3	35.3±1.8	33.2±1.8	34.6±2.1

ROM, range of motion; RN, test session after a normal reference night; CN, test session after a total sleep deprivation night; EN, test session after a total sleep deprivation night associated with 7.5 h of simulated driving (*n* = 20).

During I_1_ and I_2_, the ROM of the hip (F_(2,38)_ = 0.14; *p = *0.87 and F_(2,38)_ = 0.51; *p = *0.60 respectively), knee (F_(2,38)_ = 0.16; *p = *0.85 and F_(2,38)_ = 0.63; *p = *0.54, respectively) or ankle angles (F_(2,38)_ = 0.76; *p = *0.47 and F_(2,38)_ = 1.27; *p = *0.29) measured over the complete crank cycle did not vary significantly between the three test sessions (Table 3).

## Discussion

The aim of this study was to determine the role played by vigilance on the anaerobic performance recorded during a Wingate test performed at the bathyphase of the circadian rhythmicity. To achieve this objective, while considering the methodological constraints related to the study of the vigilance effects, a 60-s Wingate test was performed in the early morning the following day of (i) a normal reference night; (ii) a total sleep deprivation night; and (iii) a total sleep deprivation night associated with an extended simulated driving task from 9 p.m. to 5 a.m. During the Wingate test, power outputs and cycling kinetic and kinematic patterns were recorded. The main findings show that (i) after the nocturnal extended simulated driving task, the ILCs were significantly increased which demonstrates that vigilance of the participants was altered; (ii) the subjective evaluation of vigilance performed by self-rated scale revealed an increased impairment of the vigilance level between the normal reference night, the total sleep deprivation night and the total sleep deprivation night associated with an extended driving task; and (iii) the morning following this last condition, during the Wingate test, the cycling biomechanical parameters (peak power, mean power and fatigue index values, power decrease, and cycling kinetic and kinematic patterns) were not significantly different from the two other conditions.

### Body Core Temperature

GI temperatures recorded during a 24-h period prior to the testing period clearly confirm that the clock times of the morning test sessions (6 a.m.) have been set up at clock time very close to the bathyphase of the circadian rhythm in the core temperature of our participants. Indeed, they presented a circadian rhythm characterized by an acrophase at 5∶08 p.m., a bathyphase at 5∶08 a.m., and an amplitude (peak-to-trough variation) of 1°C, which is in agreement with the data found in the literature for young active participants [5]. Thus, these findings also show that the selection of the participants based on the Horne & Östberg [23] questionnaire was correct. The hours of core temperature values, which are, respectively, the lowest and the highest as observed in our study are in accordance with those found in studies performed by others in ‘‘intermediate’’-type young particpants [5], [6], [19].

### Vigilance Impairment by a Nocturnal Simulated Driving Task

Concerning the vigilance level of the participants, the use of a special fixed-based driving simulator [20] allowed us to objectively control and quantify it from the beginning to the end of the night of continuous driving. The ILCs were used to describe the driving performance, allowing us to quantify the vigilance level. The main data from the simulated driving task showed that ILCs were drastically increased during the last trip compared to the ILCs recorded during the first trip, which reveals a significant impairment in driving performance. These results are in accordance with those found by Davenne *et al.* [20] and Sagaspe *et al.* [21], which demonstrated that nocturnal driving for a long period deteriorates driving performance. The nocturnal extended driving is recognized to produce a mental fatigue-induced severe cognitive impairment that amplifies the effects of sleep deprivation on the vigilance of the driver [20], [21]. Thus, the ILCs characterize the level of vigilance and their strong impairment observed in our study is the result of an important decline in vigilance caused by both sleepiness and mental fatigue, which can be due to three main factors [20], [21]: (i) the time-of-day of driving, and/or (ii) the sleep deprivation (extended wakefulness), and/or (iii) the fatigue induced by the duration of driving. This objective evaluation was correlated with the results of the subjective sleepiness estimated throughout the nocturnal simulated driving test. They showed a strong increase between the first trip and last trip. Thus, the objective evaluation confirms the validity of the subjective evaluation of vigilance. The results of these objective and subjective parameters demonstrate that participants’ vigilance was drastically impaired during the total sleep deprivation night associated with extended simulated driving task. When comparing the self-rated sleepiness and fatigue before the Wingate test, the results reveal that the subjective sleepiness and fatigue were higher after EN than after CN and RN, and they were higher after CN than after RN, respectively.

Taken together, these findings show that the total sleep deprivation night associated with the extended simulated driving task was more disturbing than a total sleep deprivation night without driving. In addition, they confirm that the experimental task (nocturnal extended simulated driving task) proposed in this study provoked fatigue, sleepiness and an effective impairment in vigilance that was controlled and quantified.

### Anaerobic Performances after the Three Different Nights

Recordings of cycling biomechanical parameters during the sustained Wingate test revealed that PP, MP and FI values, power decrease, and kinetic (angle of peak torque and range in torque variation) and kinematic (mean angle and ROM of the hip, knee, and ankle angles) variables remained constant between the three test sessions.

Compared to a normal night, a sleep deprivation night did not affect anaerobic power output values. This finding is consistent with that obtained by Souissi *et al.* [19], which failed to show an effect of a night of total sleep deprivation on PP and MP measured during a 30-s Wingate test the following day at 6 a.m. But, in this study, the muscular fatigue phenomenon was not investigated. In our study, no effect of the total sleep deprivation night on FI and power decrease during the test was observed, which leads us to conclude that muscular fatigue, recorded during a sustained anaerobic cycling exercise, is not affected by a total sleep deprivation night. The lack of significant effect on PP, MP and FI values, and power decrease might be consistent with the fact that the main physiological factors determining the performance during a Wingate test (i.e., muscle strength of the lower limb [37]–[40] and relative contribution of anaerobic and aerobic energy systems [41]) remain unchanged after total sleep deprivation night.

Nonetheless, to be sure that there was no masking effect during the Wingate test, considering the fact that cycling movement is a complex movement that requires motor coordination based on a regulation of the different biomechanical patterns [7]–[10], a kinetic and kinematic analysis of the cycling movement was performed. To the best of our knowledge, this is the first study that examines these parameters after sleep deprivation. During the two intervals recorded during the test, kinetic and kinematic data were the same between RN and CN. These results indicate that the characteristics of cycling movement are preserved after a total sleep deprivation night. The lack of significant effect of total sleep deprivation on the biomechanical patterns would suggest that there was no sleep deprivation-induced change in the movement. It raises the question related to the effects of sleep deprivation on motor coordination which is evoked by Reilly & Deykin [42] and Shephard [43]. However, recordings of electromyographic patterns during cycling, as described, e.g., by Raasch & Zajac [9] and Wakeling & Horn [10], and Bessot *et al.* [35], are needed to test this assumption.

Interestingly, compared to the two other experimental nights (reference and control nights), the total sleep deprivation night associated with the extended simulated driving task, which induces a controlled and drastic impairment in vigilance, had no effect on the recorded performances, i.e., PP, MP and FI values, power decrease, and cycling kinetic and kinematic patterns. Different arguments can be proposed to explain this stability of the anaerobic cycling biomechanical parameters recorded during the Wingate test while vigilance level was significantly impaired. Firstly, cerebral compensatory responses might exist. They would be characterized by both recruitment of new brain regions that are not significantly involved in a given task performance in well-rested conditions, and greater activation (response) within regions typically underlying task performance [44], [45]. These compensations would contribute to counter the effects of sleep deprivation associated with vigilance impairment. Secondly, as the impairment in vigilance observed in our study at the end of a nocturnal extended simulated driving task is thought to be associated with changes in the brain system, in particular, the cortical activation level [18], the cycling movement could have been controlled at a lower level than at a cerebral system level, such as the spinal level. During cycling, the movement could be continuously modulated on the basis of control strategies employed by a low-level controller to assure the task execution [9], which would explain why this type of exercise is not sensitive to sleep deprivation associated with vigilance impairment.

### Circadian Rhythmicity

From the chronobiological point of view, it is well established that the fluctuations of the physiological and biomechanical parameters in phase with the fluctuations in the anaerobic performances recorded during the Wingate test throughout the day are related by a causal link [1], [2], [4]–[6]. Nevertheless, the results obtained in our study reveal no effect of the vigilance impairment on the data recorded during the Wingate test. These findings indicate that vigilance could fluctuate differently from anaerobic performance, which seems not to validate the assumption evoked in the literature [1], [4], [6]. According to these studies, the variations described in anaerobic power output values obtained during the Wingate test throughout the day could be related to the concomitant diurnal fluctuations in vigilance level. However, to be totally sure of that, it would be appropriate to perform several Wingate tests throughout the day. This is not possible because each Wingate test is followed by a residual fatigue. Methodologically, it would be necessary to repeat the test sessions for each point of the circadian curve.

### Conclusion

Anaerobic performance recorded during a Wingate test at the bathyphase of the circadian rhythm is not altered by total sleep deprivation night associated with an extended driving task. It means that morning anaerobic performance is not altered by vigilance impairment. These findings seem to indicate that diurnal variations in anaerobic performance would not be determined by diurnal variations in vigilance but mainly by diurnal variations in physiological and/or biomechanical parameters.
